# Evaluation of antioxidant, antibacterial and wound healing activities of *Vitex pinnata*

**DOI:** 10.12688/f1000research.21310.2

**Published:** 2020-08-07

**Authors:** Nurul Ashifah Shafie, Nur Atiqah Suhaili, Hussein Taha, Norhayati Ahmad

**Affiliations:** 1 Environmental and Life Sciences Programme, Faculty of Science, Universiti Brunei Darussalam, Jalan Tungku Link, BE1410, Brunei

**Keywords:** Vitex pinnata, antimicrobial, antioxidant, wound healing, wound excision

## Abstract

**Background** *: Vitex pinnata *is a popular ethnomedicinal plant but scientific studies to validate its pharmacological properties are lacking for this plant. **This study aims to determine the antioxidant, antimicrobial and wound healing properties of the methanolic extract of the leaves and the hexane, chloroform and ethyl acetate fractions.

**Methods:** The leaves of *Vitex pinnata* underwent methanol extraction and the methanol extract was fractionated with hexane, chloroform and ethyl acetate solvents. The antioxidant activity was determined using the DPPH radical scavenging assay. The antimicrobial activity was assessed by disc diffusion assay against *Staphylococcus aureus, Bacillus subtilis, Escherichia coli *and *Pseudomonas aeruginosa*. For the wound healing studies, the methanolic extracts of *V. pinnata *were used to prepare ointments with compositions of 10% (w/w) and 50% (w/w), which were evaluated for wound healing activity in an excision wound model in Wistar rats.

**Results:** All the extracts showed antioxidant activities, with the ethyl acetate extract having the highest DPPH radical scavenging activity, followed by the methanol, chloroform and hexane extracts. Similarly, their quercetin equivalent concentrations were 33.1, 31, 20.3 and 4.5 mg/mL, respectively. Except for the methanol extract, the disc diffusion assay showed that the extracts demonstrated species-specific antibacterial activities, with the ethyl acetate extract showing antibacterial activities against all four tested strains. The wound healing activity of the high dose treated group (50% [w/w]) shows significant increase of wound contraction when compared to the control group.

**Conclusion**: In the current study, the ethyl acetate extract showed activity for all tested bacteria and also had the highest DPPH activity. The methanolic extracts of *V. pinnata* leaves show modest wound healing activity in an excision wound model.

## Introduction

*Vitex pinnata* is a common ethnomedicinal plant locally known as ‘kulimpapa’ in Brunei Darussalam and East Malaysia. According to Goh *et al.* (2017), it is traditionally used as a treatment for stomach aches, fever, body aches and lesions. The bark may be used for abdominal pain, while the young shoots are utilized as sanitizing agents and deodorants. The roots and bark of this plant have been used in herbal baths and also taken orally in the form of decoction or herbal tea while the leaves may be eaten raw. Traditionally, the leaves were prepared into poultice and applied to wounds to induce fast healing ( Burkill *et al.,* 1966; Goh *et al.,* 2017; Sahu & Barik, 2007; Suksamrarn & Sommechai, 1993). Although *V. pinnata* is a popular ethnomedicinal plant, scientific studies are lacking to validate its pharmacological properties. It is not clear if this plant has antioxidant, antimicrobial and wound healing properties.

Plants have been known to be a potent source of medicinal compounds and anti-oxidants. Oxidative stress produced through a chain reaction by free radicals can be a contributing factor to the pathophysiology of various conditions including cardiovascular dysfunction, atherosclerosis, inflammation, carcinogenesis, reperfusion injury and neurodegenerative diseases ( Aruoma, 1998). Due to the increasing safety concerns with the consumption of synthetic antioxidants, alternative sources of antioxidants from natural origins, especially from plants, are currently in demand ( Stankovic *et al.,* 2016). This study is interested to determine if *V. pinnata* could be a good source of antioxidants.

Although various antibacterial agents of synthetic origin are available, bacterial resistance to current antibacterial agents is growing ( Andersson & Hughes, 2010). Furthermore, available antibiotics can also cause side effects. For this purpose, plants can be good sources of antimicrobial agents due to their secondary metabolites ( Nascimento *et al.,* 2000). Previous studies have reported the antifungal activity of *V. pinnata* ( Ata *et al.,* 2009), however its antibacterial and antioxidant activity has not yet been reported. Therefore, this study also aims to screen its antibacterial activity against several bacterial strains.

In order to understand the possible effects of *Vitex pinnata* on wound healing, we have sought the use of animal models to reflect human wound healing conditions. Wistar rats were selected in our study because of their availability. In addition to this, the use of Wistar rats in this study provides a more mainstream model for understanding the wound healing process upon treatment with the extract. The use of this species and excision wound model also allows us to make a comparison of the wound healing process with other available literature involving a similar approach to ours. The scientific objective of this study is to determine the stage by stage process of wound healing such as general wound morphology, wound closure and presence of inflammatory cells, which could only be carried out in an animal model.

In this study, we report the antibacterial, antioxidant and wound healing activities of *V. pinnata* from Brunei Darussalam.

## Methods

### Chemicals and reagents

Ethyl acetate, chloroform, hexane, methanol and Xylene were purchased from Merck, Germany. DPPH (2,2-diphenyl-1-picrylhydrazyl), quercetin and streptomycin sulphate were from Sigma-Aldrich, Germany. Nutrient broth and Mueller-Hinton agar (MHA) were from Oxoid, United Kingdom. Diethyl ether was obtained from Emsure®, Germany. Formaldehyde was obtained from Scharlau, Spain. Paraffin wax was from Thermo scientific, United Kingdom. Hematoxylin was obtained from Fluka, Switzerland.

### Plant materials and preparation of extracts

The leaves of *V. pinnata* were collected at the Universiti Brunei Darussalam Botanical Research Centre (UBD BRC) in Brunei Darussalam. Species identification was kindly carried out by the botanist at the UBD BRC. Voucher specimen of *V. pinnata* (catalog ID S00059) is available in UBD Herbarium ( http://ubdherbarium.fos.ubd.edu.bn/). The plant samples were shade dried for a few weeks and grinded prior to solvent extraction.

A mass of 300 g of the ground leaves was exhaustively extracted with 1.5 L of methanol using Soxhlet extraction. The resulting methanol extract was then vacuum filtered and evaporated using a rotary evaporator. The methanol extract was further partitioned with different solvents in increasing order of polarity i.e. hexane, chloroform and ethyl acetate. A total of 10 g of the methanol extract was redissolved in approximately 1 L of methanol and 10 mL of distilled water, followed by the addition of 500 mL of hexane. After shaking vigorously, the mixture was left to stand to allow layers to form between the solvents. Once formed, the hexane layer was collected and the procedure was repeated with the other solvents (chloroform and ethyl acetate). Each solvent was evaporated with a rotary evaporator and subsequently air dried in a fume hood.

The percentage yield of extract was determined using the formula: Extraction yield (%) = [Dry weight of extract obtained / Dry weight of material used for extraction] × 100%. Prior to the antioxidant and antibacterial tests, each extract was re-dissolved in methanol at 1000 μg/mL and 500 mg/mL, respectively.

### DPPH radical scavenging assay

The antioxidant or radical scavenging activity (RSA) was determined by DPPH radical scavenging assay according to Awang-Jamil *et al.* (2019) with minor modifications. Each extract with a volume of 0.2 mL was mixed with 1 mL of 40 μg/mL of DPPH methanolic solution. For the control, 0.2 mL of methanol was used instead of the extract. After about 30 minutes, the RSA was determined by measuring the absorbance at 517 nm using a UV spectrophotometer. The assay was carried out in triplicate. The RSA (measured as the percentage of DPPH scavenged) was calculated using the formula: RSA (%) = [A _control_ – A _sample_]–/A _control_ ** × 100%, where A _control_ refers to the absorbance of the control and A _sample_ refers to the absorbance of the extract.

To measure the quercetin equivalent (QE) concentration of each extract, the RSA of quercetin at six different concentrations (1, 5, 10, 20, 25 and 35 µg/mL) were also similarly measured. A standard calibration curve was prepared by plotting RSA (y-axis) against quercetin concentration (x-axis). Subsequently, the linear regression of the standard calibration curve (y = 2.74x + 2.77; R ^2^ = 0.997) was employed for the estimation of QE concentration.

### Antibacterial assay

The microorganisms used in this study consisted of four different strains of bacteria: two Gram-positive bacteria, *Staphylococcus aureus* (ATCC-29213) and *Bacillus subtilis* (ATCC-11774); and two Gram-negative bacteria, *Escherichia coli* (ATCC-11775) and *Pseudomonas aeruginosa* (ATCC-27853). Each bacterial strain was grown in nutrient broth, and prior to the test, overnight bacterial culture was diluted to an absorbance of 0.08 to 0.1 (equivalent to 0.5 McFarland standard), measured with a UV spectrophotometer at 600 nm.

Antibacterial activities of the extracts were determined by disc diffusion method according to Abdullah *et al.* (2019) with minor modifications. A volume of 10 µl of each extract was applied onto a filter paper disc (6 mm in diameter) and left to dry. Methanol was used as the negative control and 20 mg/ml streptomycin sulphate (antibiotic) as the positive control. Each standardised bacterial culture was then swabbed onto Mueller-Hinton agar (MHA) plates. The impregnated discs were then loaded onto the swabbed MHA plates. The zone of inhibition was recorded after incubating the plates at 37 °C for 24 hours. Each test was carried out with at least three replicates.

### Wound healing activity

***Ethical statement.*** All work involving animals was approved by the Universiti Brunei Darussalam University Research Ethics Committee [approval reference: UBD/FOS/E2(g)]. All animal procedures were performed in accordance with Universiti Brunei Darussalam guidelines on the care and use of animals for research and internationally accepted guidelines. All investigators declare that every effort was taken to ameliorate harm to the animals via close monitoring for signs of pain and distress, and attended to in case signs of pain and distress was observed in accordance with the Universiti Brunei Darussalam guideline on care and use of animals for research.

***Animals.*** Male Wistar rats aged between 8–10 weeks and weight range between 150–200g were used for the wound healing study. The animals were obtained from and housed in the Universiti Brunei Darussalam animal facility. The rats were fed with a standard maintenance pellet diet (Cat # 1324, Altromin, Germany) and access to food and water was provided *ad libitum*. Animals were housed in cages with wood shavings as bedding and maintained under standard lab conditions (12/12h light dark cycle; 25°–30°C). Prior to carrying out the experimental procedures, animals were assessed for their general health, determined by their behavioral responses to the handler and other general parameters such as hands-on physical examination, condition of the animal’s body, physical and observable abnormalities. The welfare of the animals was attended to throughout the experiments such as by the provision of food and water, avoidance by the handler of conditions that cause suffering, pain and disease. Animals with obvious signs of pain and distress during the experiment would be excluded from the study.

Control animals were placed between three to four animals per cage and following the wound excision procedure, animals were placed in individual cages throughout the duration of the experiment. Upon completion of the experiments, animals were euthanized using CO _2_ asphyxiation followed by cervical dislocation prior to tissue collections for histological analysis.

***Formulation of ointment.*** The methanolic leaf extracts of *V. pinnata* were formulated into ointments with pure petroletum jelly as the base. Herbal ointments were prepared in two doses, which is the low dose extract consisting of 10% (w/w) extract and high dose extract containing 50% (w/w) extract. The maximum concentration that was stable in the base was 50% (w/w).

***Excision wound.*** All procedures were refined in order to minimize any negative impact and pain to the animals resulting from the excision wound procedure. All procedures were carried out in the Universiti Brunei Darussalam animal facility at the same time of day between 10.00–13.00hrs. Prior to making the excision wound, all treated animals were anaesthetized using diethyl ether by inhalation anesthesia. Diethyl ether was chosen as the anesthetic because its action is also accompanied by analgesic properties. Animals were exposed to one drop of diethyl ether placed in a cotton wool and placed at one end of a conical tube. There was no close or direct contact between the cotton ball and the nose of the animal. Depth of anesthesia following exposure to diethyl ether was determined by responses to reflexes such as voluntary movements from stimuli such as extending of legs. Once deep anesthesia has been established, the animal excision wounds were performed as previously described ( Umachigi *et al.,* 2008). The dorsal fur of the animals was shaved and an area of about 100mm2 (10 × 10 mm) was excised using a sharp scalpel with disposable steel blades. Rats were then individually housed and the extract and ointment were applied topically on the wound area at the same time of day (between 10am –1pm) in the respective groups for a period of 21 days.

***Animal grouping and dosing.*** Animals were randomly grouped into three groups, each group containing six rats. The numbers of animals (n) used in these experiments were based on previous studies in our laboratory and experiments of this nature ( Nagar *et al.,* 2016) and is also the minimum that is required to detect any significant difference between the treatment groups. Simple randomization was carried out when assigning the animals to groups in order to avoid any bias. Introduction of bias is reduced or eliminated as during the early stages of the experiment; i.e. during grouping, allocation of animals to treated and control groups was carried out randomly. Wound area determination was carried out by a validated instrument (Image J software, National Institute of Health, USA) which minimizes subjectivity of observer during assessment of wound areas.

Following this, all animals within each group received the same treatment throughout the duration of the experiment. The rats of Group I were treated with pure petroletum jelly only (negative control). Group 2 and Group 3 were treated with ointments containing 10% (w/w) leaf extract (low dose) and 50% (w/w) leaf extract (high dose), respectively. The ointment was topically applied to the wounds on alternate days for a period of three weeks.

**Figure 1.**
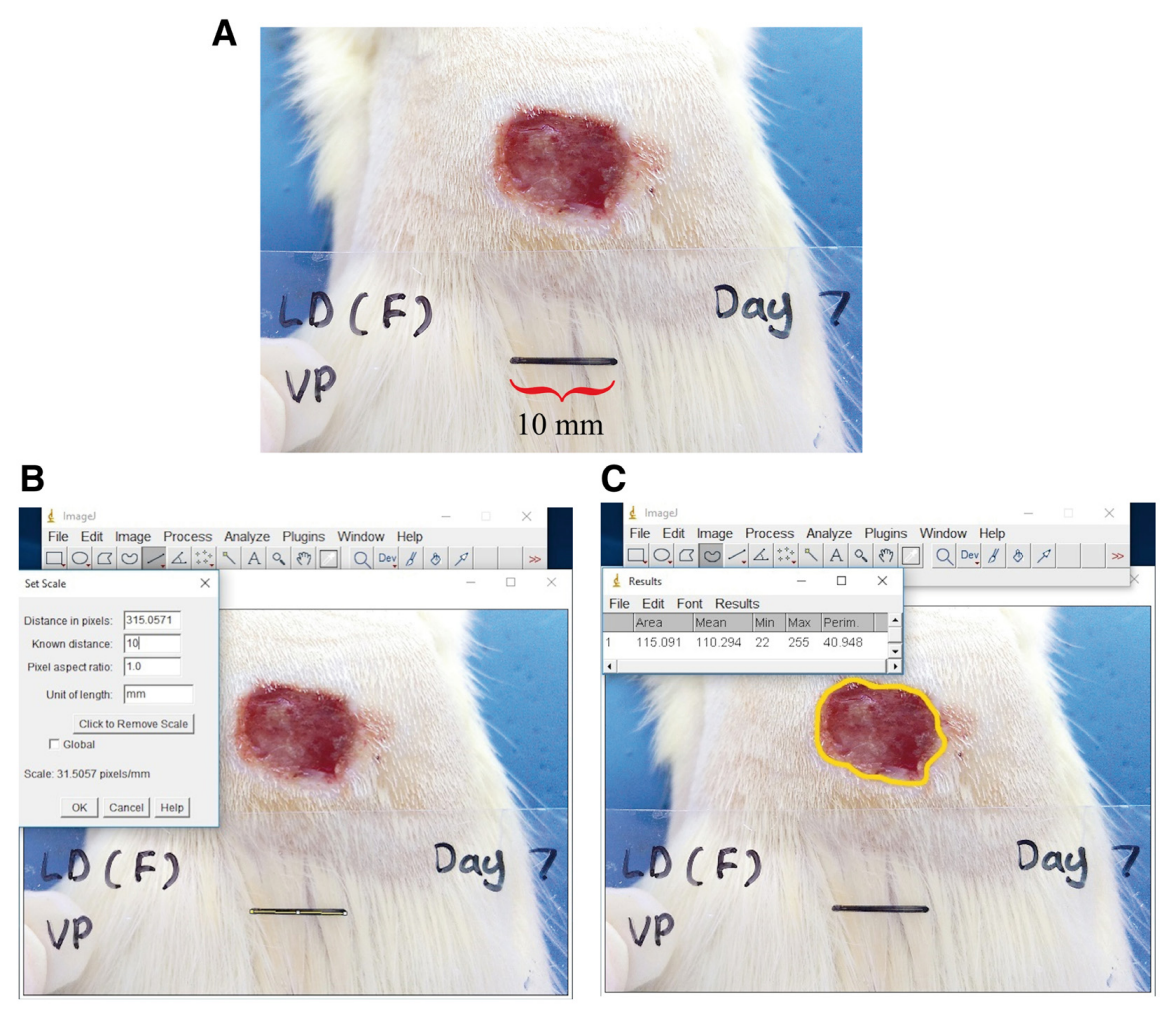
**A**) Anaesthetized rat with the scale placed near the wound site, labelled with name, post-wounding day and scale bar of 10 mm for measurement analysis. **B**) Calibration of Image J with known distance in image. **C**) The outline of the wound is drawn to measure wound area.

***Wound area determination.*** Wound area was measured using Image J software (National Institute Health, USA) ( Figure 1). The rate of wound contraction was measured as the percentage of reduction of wound size. The percentage of wound contractions were calculated as previously described ( Shi *et al.,* 2013): Wound contraction (%) = 100 × [(Original wound area – area on measured post-wounding day) / Original wound area]

***Histological analysis.*** Skin tissue samples from the wound and its vicinity were taken for histopathological analysis at post wounding days 3, 7, 14 and 21. Sections at 10µm thickness were prepared and stained with hematoxylin and visualized.

### Statistical analysis

Statistical analyses were conducted with one-way ANOVA and a post hoc Tukey’s HSD test. These were performed using R 3.3.3 software. Values of p < 0.05 indicated statistical significance.

## Results and discussion

### Extraction yields

Soxhlet extraction yielded 8.0% of methanol extract from 300 g of ground leaves. For solvent partitioning, 20.3% hexane, 62.1% chloroform and 8.9% ethyl acetate extracts were obtained from 10 g of methanol extract.

### Antioxidant activities

Statistical analysis indicated that the RSA of the different extracts were significantly different to each other. The ethyl acetate extract had the highest RSA and QE concentration, indicating it was the most potent in inhibiting free radicals compared to the other extracts ( Table 1). This was followed by the methanol extract, the chloroform extract and finally the hexane extract.

**Table 1.** Radical scavenging activities (RSA) and quercetin equivalent (QE) concentrations of *V. pinnata* extracts.

Extract	RSA (%)	QE concentration (µg/mL)
Ethyl acetate	93.6 ± 0.2	33.1 ± 0.1
Methanol	87.8 ± 0.5	31 ± 0.2
Chloroform	58.5 ± 1.5	20.3 ± 0.6
Hexane	15.1 ± 1.4	4.5 ± 0.5

The values are shown as average ± standard deviation of three replicates.

Different solvents used for extraction have been reported to extract different compounds ( Basri *et al.,* 2017). It has been previously reported that phenolic content increased with the increasing polarity of solvent ( Barchan *et al.,* 2014; Belyagoubi *et al.,* 2016). The extracts from *V. pinnata* in the present study possibly contained different types of phenolic compounds with different antioxidant capacities. Zhang (2015) has studied the effects of solvents on the phytochemical contents. It was found that the extraction of total flavonoids significantly increased in polar solvents, which might contribute to the antioxidant activity in *V. pinnata*. However, in this study, ethyl acetate had higher antioxidant activity compared to the more polar methanol. This could probably mean that certain non-polar compounds, which probably dissolved better in ethyl acetate, might also contribute to the higher antioxidant activity.

In the present study, the ethyl acetate and hexane extract showed the highest and lowest radical scavenging activities amongst the extracts, respectively. Similarly, a previous study conducted by Vats (2012) on cowpea ( *Vigna unguiculata*) also showed that ethyl acetate extract had the best DPPH radical scavenging activity. Moreover, the highest phenolic content was also found in the ethyl acetate extract, implying that most phenols were soluble in ethyl acetate. The lowest scavenging activity was in the hexane extract, which could be attributed to its low amount of phenolic content.

Closely related plant species have been shown to have similar phytochemical constituents ( Diris *et al.,* 2017). The antioxidant activities of *V. pinnata*’s close relatives, *V. agnus-cactus*, *V. negundo* and *V. trifolia* have also been determined using various solvents, and was found that the fruits and leaves of these *Vitex* plants ** were significantly capable of scavenging free radicals ( Rashed, 2013; Saklani *et al.,* 2017), signifying that *Vitex* plants are potential sources of promising antioxidants.

### Antibacterial activity

Detected zones of inhibition as shown in Table 2 indicated the presence of antibacterial activities in the extracts of *V. pinnata* leaves at 500 mg/mL.

**Table 2.** Zone of inhibition (mm) of *V. pinnata* extracts.

Zone of inhibition (mm)				
Bacteria	Methanol	Hexane	Chloroform	Ethyl acetate
*E. coli*	0.0 ± 0.0	0.0 ± 0.0	8.4 ± 0.1	10.2 ± 0.3
*B. subtilis*	0.0 ± 0.0	8.2 ± 1.2	9.3 ± 0.1	9.3 ± 0.5
*P. aeruginosa*	0.0 ± 0.0	8.0 ± 0.4	10.3 ± 0.1	11.6 ± 0.2
*S. aureus*	0.0 ± 0.0	0.0 ± 0.0	0.0 ± 0.0	6.2 ± 0.5

The values are shown as average ± standard deviation of at least three replicates. Negative control did not show any inhibition zone as expected whereas positive control (streptomycin sulphate) showed inhibition zones ranging from ~20 to 30 mm.

The results generally showed the presence of antibacterial activities in the hexane, chloroform and ethyl acetate extracts and no antibacterial activity detected in the methanol extract. As shown in Table 2, the ethyl acetate extract could inhibit all the bacterial strains as compared to chloroform extract, which could only inhibit a total of three bacterial strains, and hexane extract, which could inhibit two strains.

### Wound healing

**Figure 2.**
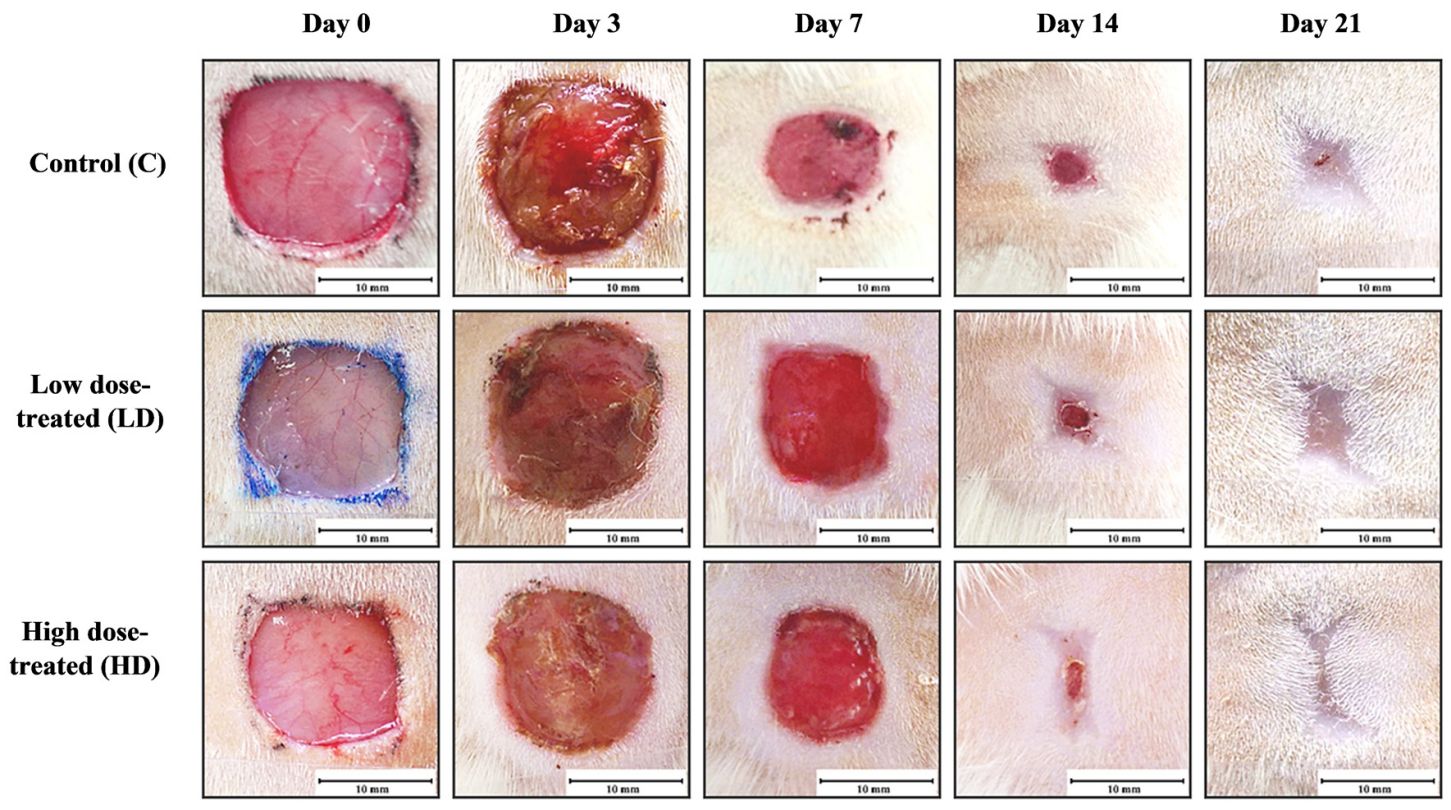
Macroscopic observation of wound healing progress in control and extract-treated groups at given post-wounding time points.

**Figure 3.**
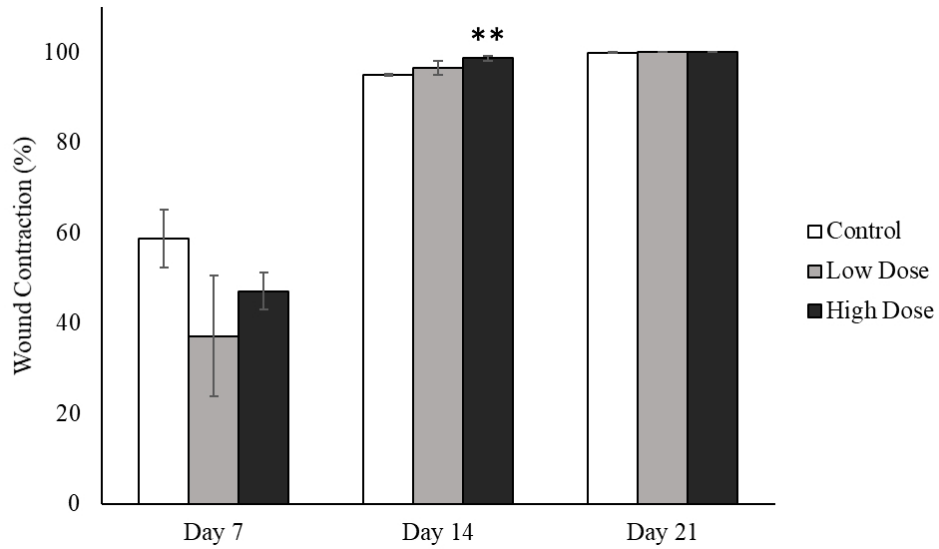
Average percentages of wound contraction of the experimental groups at post-wounding days 7, 14 and 21 (n= 3). ** denote significant changes of wound contraction at day 14 in comparison to control group, **p < 0.01.

All animals were continuously observed and assessed for their general health status throughout the duration of the experiment. We did not observe any adverse effects in these animals such as infections of the excised region or any behavioral changes following the excision wounding and during the recovery period. The difference in wound area was observed from post-wounding day 7 in all experimental groups ( Figure 2). The wound contraction at post-wounding day 7 was determined to be slightly higher for animals in the negative control group when compared to extract-treated groups ( Figure 3). At post-wounding day 14, wound contraction in extract-treated groups were considerably higher when compared to the control group and particularly in animals receiving the higher concentration of extract ( Table 3). Complete wound closure was observed in both the low dose 10% (w/w) and high dose 50% (w/w) extract treated groups at post-wounding day 21, indicating a faster rate of epithelialization compared to normal wound healing in the control group.

**Table 3.** Percentage wound contraction for animals receiving low and high dose extracts vs controls.

Groups	Wound contraction (%)			
	Day 3	Day 7	Day 14	Day 21
**Control**	2.4 ± 10.1	58.8 ± 6.4	95.0 ± 0.3	99.9 ± 0.1
**Low dose**	-14.4 ± 19.1	37.1 ± 13.4	96.6 ± 1.6	100 ± 0.0
**High** **dose**	-4.2 ± 8.5	47.1 ± 4.1	98.7 ± 0.6**	100 ± 0.0

Values are mean ± SEM; **p < 0.01 compared to control animals on similar treatment days. The starting experiment had n=6 animals per group. One animal was sacrificed for histological analysis at day 3, day 7 and day 14. There was n=3 animals remaining at day 21 which is the final day of the experiment.

Our study demonstrated a dose dependent effect of the methanolic extract on wound healing, as evident from the wound closure observed for the 10% (w/w) vs 50% (w/w) extract treated animals. General wound morphology in post wounding day 3 shows a well-developed scab formation in all groups ( Figure 2). We did not observe any adverse effects in both control and extract treated animals. At day 14, there was almost complete closure of the wound in the high dose group ( Figure 2).

**Figure 4.**
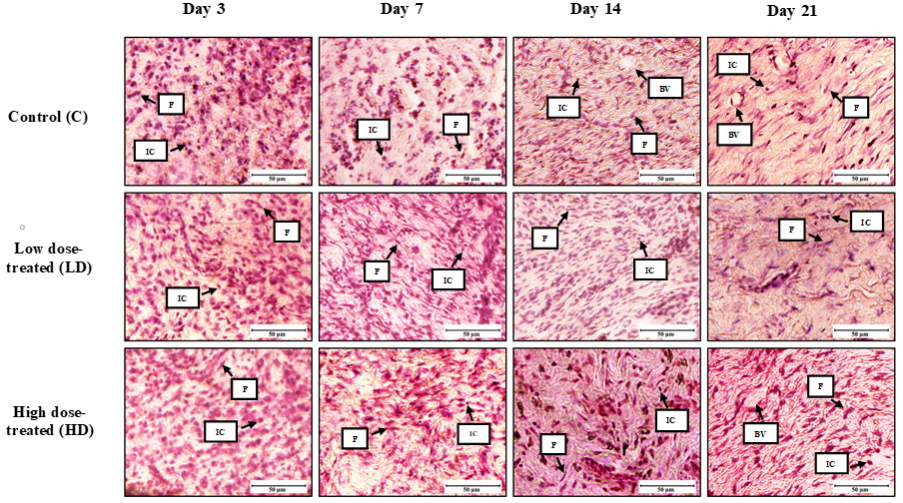
Histopathological characteristics of wound granulation tissue on post-wounding days 7, 14 and 21 stained with hematoxylin. Day 3 control and treated rats: presence of inflammatory cells is evident. Collagen deposition observed in the high dose treated animals at day 14. BV: blood vessels; F: fibroblasts; IC: inflammatory cells (x400).

We observed a high density of inflammatory cells in the low dose and high dose treated tissues ( Figure 4). The low dose and high dose treated animals showed epithelialization at day 7, whereas the control group showed epithelialization at day 14. Inflammatory cells become prominent, which may include neutrophils, followed by macrophages and mast cells that emigrate from nearby tissues, playing a crucial role in combating infection ( Martin & Leibovich, 2005).

Based on histological observations, granulation tissue from all experimental groups displayed increased infiltration of fibroblast cells, indicative of active proliferation ( Figure 4). Among all of the experimental groups, high-dose treated group showed greater wound contraction at post-wounding day 14 ( Table 3). At post-wounding day 21, the wound in the high-dose treated group showed a more densely packed organization of collagen and connective tissues compared to the low dose-treated and control group.

Based on the wound contraction evaluation and histological analysis of the wound, our study has demonstrated a modest wound healing effect in the *V. pinnata* high-dose treated group followed by low-dose treated group as compared to control group ( Figure 2). Phytochemical analysis reports have revealed that the petroleum ether, ethyl acetate, methanol and aqueous leaf extracts of *V. pinnata* contain varying amounts of the alkaloid, anthocyanidins, aucubins, coumarins, flavonoids, flavanols, gallic tannins, iridoids, proteins, reducing compounds, steroids, triterpenoids and glycoside compounds, of which flavonoids appeared to be present in the highest quantity in all the four different extracts ( Ramesh *et al.,* 2013). The chemical constituents of its barks have also been reported to include flavonoids ( Ata *et al.,* 2009). These phytochemicals are known to possess antioxidant, anti-inflammatory, and anti-microbial properties which are responsible for the enhanced rate of wound healing observed ( Shah & Amini-Nik, 2017).

## Conclusion

The extracts from *V. pinnata* leaves showed the ability to scavenge free radicals, with the ethyl acetate extract being the most potent. All extracts except for the methanol extract also exhibit species-specific antibacterial activities, with the ethyl acetate extract also being the most potent. Modest wound healing capabilities are also observed in the *V. pinnata* treated excision wound. Study limitations include our inability to determine the relative absorption of the plant extract into the excision wound tissue site, which may have an effect on the wound closure. Further optimization of the plant extract also needs to be carried out to elucidate factors such as toxicity and effective doses for human use.

We have focused on the effects of *V. pinnata* extract on an excision wound, the study could be extended to include other wound models such as burns and incision wounds in order to ascertain the plant extract’s wound healing capabilities.

## Data availability

### Underlying data

Figshare: Evaluation of antioxidant, antibacterial and wound healing activities of Vitex pinnata. https://doi.org/10.6084/m9.figshare.11369973.v1 ( Shafie *et al.,* 2019)

Data are available under the terms of the Creative Commons Attribution 4.0 International license (CC-BY 4.0).

## References

[ref-1] Abdullah NA Ja'afar F Yasin HM: Physicochemical analyses, antioxidant, antibacterial, and toxicity of propolis particles produced by stingless bee *Heterotrigona itama*found in Brunei Darussalam. *Heliyon.* 2019;5(9):e02476. 10.1016/j.heliyon.2019.e02476 31687571 PMC6819780

[ref-2] Andersson DI Hughes D: Antibiotic resistance and its cost: is it possible to reverse resistance? *Nat Rev Microbiol.* 2010;8(4):260–271. 10.1038/nrmicro2319 20208551

[ref-4] Aruoma OI: Free radicals, oxidative stress, and antioxidants in human health and disease. *J Am Oil Chemist's Soc.* 1998;75(2):199–212. 10.1007/s11746-998-0032-9 PMC7101596 32287334

[ref-5] Ata A Mbong N Iverson CD: Minor chemical constituents of *Vitex pinnata.* *Nat Prod Commun.* 2009;4(1):1–4. 10.1177/1934578X0900400102 19370864

[ref-6] Awang-Jamil Z Basri AM Ahmad N: Phytochemical analysis, antimicrobial and antioxidant activities of *Aidia borneensis* leaf extracts. *J Appl Biol Biot.* 2019;7(5):92–97. 10.7324/JABB.2019.70515

[ref-7] Barchan A Bakkali M Arakrak A: The effects of solvents polarity on the phenolic contents and antioxidant activity of three *Mentha* species extracts. *Int J Curr Microbiol Appl Sci.* 2014;3(11):399–412. Reference Source

[ref-8] Basri AM Taha H Ahmad N: A review on the pharmacological activities and phytochemicals of *Alpinia officinarum* (Galangal) extracts derived from bioassay-guided fractionation and isolation. *Pharmacogn Rev.* 2017;11(21):43–56. 10.4103/phrev.phrev_55_16 28503054 PMC5414456

[ref-9] Belyagoubi L Belyagoubi-Benhammou N Coustard JM: Effects of extraction solvents on phenolic content and antioxidant properties of *Pistacia atlantica.*Desf fruits from Algeria. *Int Food Res J.* 2016;23(3):948–953. Reference Source

[ref-11] Burkill I Birtwistle W Foxworthy F: A dictionary of the Economic Products of the Malay Peninsula (2nd ed.).Kuala Lumpur: Ministry of Agriculture and Co-operatives, Kuala Lumpur.1966 Reference Source

[ref-18] Diris MN Basri AM Metali F: Phytochemicals and antimicrobial activities of *Melastoma malabathricum* and *Melastoma beccarianum* leaf crude extracts. *Res J Phytochemistry.* 2017;11(1):35–41. 10.3923/rjphyto.2017.35.41

[ref-21] Goh MP Basri AM Yasin H: Ethnobotanical review and pharmacological properties of selected medicinal plants in Brunei Darussalam: *Litsea elliptica*, *Dillenia suffruticosa*, *Dillenia excelsa*, *Aidia racemosa*, *Vitex pinnata* and *Senna alata*. *Asian Pac J Trop Biomed.* 2017;7(2):173–180. 10.1016/j.apjtb.2016.11.026

[ref-26] Martin P Leibovich SJ: Inflammatory cells during wound repair: the good, the bad and the ugly. *Trends Cell Biol.* 2005;15(11):599–607. 10.1016/j.tcb.2005.09.002 16202600

[ref-27] Nagar HK Srivastava AK Srivastava R: Pharmacological Investigation of the Wound Healing Activity of *Cestrum nocturnum* (L.) Ointment in Wistar Albino Rats. *J Pharm (Cairo).* 2016;2016: 9249040. 10.1155/2016/9249040 27018126 PMC4785265

[ref-28] Nascimento GG Locatelli J Freitas PC: Antibacterial activity of plant extracts and phytochemicals on antibiotic-resistant bacteria. *Brazilian J Microbiol.* 2000;31(4):247–256. 10.1590/S1517-83822000000400003

[ref-31] Ramesh S Rajasekar K Venkata Raju RR: Preliminary phytochemical studies on leaves of *Vitex* species (Verbenaceae), used by the local adivasi communities of Andhra Pradesh. *World J Pharm Pharm Sci.* 2013;2:6143–6150. Reference Source

[ref-32] Rashed KN: Antioxidant activity of different extracts of *Vitex agnus-castus* (L.) and phytochemical profile. *Res Pharm.* 2013;3(6):1–5. Reference Source

[ref-37] Sahu P Barik B: Antipyretic and wound healing activity of aqueous extract of leaves of Vitex pinnata. *Indian Drugs.* 2007;44(7):532–534. Reference Source

[ref-38] Saklani S Mishra AP Chandra H: Comparative evaluation of polyphenol contents and antioxidant activities between ethanol extracts of *Vitex negundo* and *Vitex trifolia* L. leaves by different methods. *Plants (Basel).* 2017;6(4): pii: E45. 10.3390/plants6040045 28953235 PMC5750621

[ref-40] Shafie NA Suhaili NA Taha H: Evaluation of antioxidant, antibacterial and wound healing activities of Vitex pinnata. *figshare.*Dataset.2019 10.6084/m9.figshare.11369973.v1 PMC7445684 32874549

[ref-41] Shah A Amini-Nik S: The Role of Phytochemicals in the Inflammatory Phase of Wound Healing. *Int J Mol Sci.* 2017;18(5): pii: E1068. 10.3390/ijms18051068 28509885 PMC5454978

[ref-42] Shi HX Lin C Lin BB: The anti-scar effects of basic fibroblast growth factor on the wound repair *in vitro* and *in vivo*. *PLoS One.* 2013;8(4):e59966. 10.1371/journal.pone.0059966 23565178 PMC3615060

[ref-43] Stankovic N Mihajilov-Krstev T Zlatkovic B: Antibacterial and antioxidant activity of traditional medicinal plants from the Balkan Peninsula. *NJAS-Wagen J Life Sc.* 2016;78:21–28. 10.1016/j.njas.2015.12.006

[ref-44] Suksamrarn A Sommechai C: Ecdysteroids From *Vitex pinnata*. *Phytochemistry.* 1993;32(2):303–306. 10.1016/S0031-9422(00)94985-9

[ref-46] Umachigi SP Jayaveera KN Ashok Kumar CK: Studies on wound healing properties of *Quercus infectoria*. *Trop J Pharm Res.* 2008;7(1):913–919. 10.4314/tjpr.v7i1.14677

[ref-47] Vats S: Antioxidant activity of callus culture of *Vigna unguiculata* (L.) Walp. *Researcher.* 2012;4(6):22–24. Reference Source

[ref-49] Zhang Q: Effects of extraction solvents on phytochemicals and antioxidant activities of walnut ( *Juglans regia* L.) green husk extracts. *Eur J Food Sci Tech.* 2015;3(5):15–21. Reference Source

