# Supplementary material for: Evaluation of antioxidant, antibacterial and wound healing activities of Vitex pinnata
Source: F1000Res. 2020 Aug 7;9:187. Originally published 2020 Mar 13. [Version 2] doi: 10.12688/f1000research.21310.2 (PMC7445684; doi:10.12688/f1000research.21310.2)
Supplement: Peer review history

# Peer review: Reviewer response for version 2

**DOI:** 10.5256/f1000research.28592.r69006

**Published:** 2020-09-07

**Authors:** Fitmawati Fitmawati

**Affiliations:** 1 Department of Biology, Faculty of Mathematics and Natural Sciences, Riau University, Riau, Indonesia

The author has improved and written the improvements as suggested, so there are no further comments.

Is the work clearly and accurately presented and does it cite the current literature?

Yes

If applicable, is the statistical analysis and its interpretation appropriate?

Yes

Are all the source data underlying the results available to ensure full reproducibility?

Yes

Is the study design appropriate and is the work technically sound?

Yes

Are the conclusions drawn adequately supported by the results?

Yes

Are sufficient details of methods and analysis provided to allow replication by others?

Yes

Reviewer Expertise:

Herbal Medicine, Plant Taxonomy

I confirm that I have read this submission and believe that I have an appropriate level of expertise to confirm that it is of an acceptable scientific standard.

# Peer review: Reviewer response for version 2

**DOI:** 10.5256/f1000research.28592.r69005

**Published:** 2020-08-24

**Authors:** Yoke-Keong Yong

**Affiliations:** 1 Department of Human Anatomy, Faculty of Medicine and Health Sciences, Universiti Putra Malaysia, Serdang, Malaysia

Overall, the authors did the correction according to the comments and suggestions. However, I have another one more question on wound healing assay. Why only methanolic extract tested for wound healing since ethyl acetate extract showed activity for all tested bacteria and also had the highest DPPH activity? Anti-bacteria and antioxidant contribute greatly in wound healing process, so why ethyl acetate extract has not tested for wound healing in this case?

Is the work clearly and accurately presented and does it cite the current literature?

Partly

If applicable, is the statistical analysis and its interpretation appropriate?

No

Are all the source data underlying the results available to ensure full reproducibility?

Yes

Is the study design appropriate and is the work technically sound?

Yes

Are the conclusions drawn adequately supported by the results?

Yes

Are sufficient details of methods and analysis provided to allow replication by others?

Partly

Reviewer Expertise:

Natural products, Complementary and alternative medicine, vascular biology, cancer biology, non-communicable diseases.

I confirm that I have read this submission and believe that I have an appropriate level of expertise to confirm that it is of an acceptable scientific standard.

# Peer review: Reviewer response for version 1

**DOI:** 10.5256/f1000research.23467.r67298

**Published:** 2020-07-20

**Authors:** Fitmawati Fitmawati

**Affiliations:** 1 Department of Biology, Faculty of Mathematics and Natural Sciences, Riau University, Riau, Indonesia

Generally, this article provides novelty information in the field of natural antioxidant sources from the tropics. The method used is good and detailed. The results have been explained well, as well as the discussion. However, the abstract did not convey a compelling reason to underlie this study, so the impression of this study was flat. I suggest writing three sentences that show the reasons for the importance of this research in the abstract and in the introduction. Then, explain fluently the strong relationship between the elements of the title, background, objectives, methods, results, and conclusions. 

 For example abstract :

*Vitex pinnata* is one of the many plants known for its ethnomedicinal properties (choose the strong sentence for the first to give a strong reason in choosing this research).

 In the same case in the introduction, make sure the word chosen is a strong word to underlie this research and is described logically and fluently.

Is the work clearly and accurately presented and does it cite the current literature?

Yes

If applicable, is the statistical analysis and its interpretation appropriate?

Yes

Are all the source data underlying the results available to ensure full reproducibility?

Yes

Is the study design appropriate and is the work technically sound?

Yes

Are the conclusions drawn adequately supported by the results?

Yes

Are sufficient details of methods and analysis provided to allow replication by others?

Yes

Reviewer Expertise:

Herbal Medicine, Plant Taxonomy

I confirm that I have read this submission and believe that I have an appropriate level of expertise to confirm that it is of an acceptable scientific standard.

# Response

**Published:** 2020-08-04

**Authors:** NORHAYATI AHMAD

**Affiliations:** UNIVERSITI BRUNEI DARUSSALAM, Brunei

Many thanks to the reviewer for kindly reviewing our manuscript. We have changed the abstract to: “ *Vitex pinnata* is a popular ethnomedicinal plant but scientific studies to validate its pharmacological properties are lacking for this plant.”

We also have revised the introduction by adding the sentences below:

- Although *V. pinnata* is a popular ethnomedicinal plant, scientific studies are lacking to validate its pharmacological properties. It is not clear if this plant has antioxidant, antimicrobial and wound healing properties.
- This study is interested to determine if *V. pinnata* could be a good source of antioxidants.  
- Therefore, this study also aims to screen its antibacterial activity against several bacterial strains.

# Peer review: Reviewer response for version 1

**DOI:** 10.5256/f1000research.23467.r63436

**Published:** 2020-05-20

**Authors:** Yoke-Keong Yong

**Affiliations:** 1 Department of Human Anatomy, Faculty of Medicine and Health Sciences, Universiti Putra Malaysia, Serdang, Malaysia

**Section: Introduction**

- Problem statement not clear. 

**Section: Methods**

- Suggest to add another section on “chemicals & reagents”, the authors should list all the chemicals and reagents used in this study by clearly stating the brand, company, and country. 

 

**Section: Plant materials and preparation of extracts**

- Kindly provide a voucher specimen of the plant.

 

**Section: Results and discussion**

- Kindly provide yield results.
- Caption for all the results (Table and Figure incomplete).

 

**Section: Results and discussion – Antioxidant activities**

- Authors stated and cited phenolic content increased with the increasing polarity of solvent and extraction of total flavonoids significantly increased in polar solvents, which might contribute to the antioxidant activity by Barchan *et al.*, 2014 and Zhang, 2015. However, in the current study ethyl acetate showed to have higher antioxidant activities compared to methanol where methanol polarity is higher than the ethyl acetate. Kindly justify this.

 

**Section: Results and discussion – Antibacterial activity**

- The authors should include the results for a positive control (reference drug), as it enables a comparison with the other treatment group.
- In addition, there is no statistical analysis provided.
- The concentration of the reference drug should be provided.

 

**Section: Results and discussion – Wound healing**

- Kindly provide details of the figures and tables, such as SEM or SD.
- Kindly re-run the statistical analysis for the data of Figure 3 (especially Day 14) as the Control group showed big SEM or SD, however, there was a significant difference comparing Control and High dose at P<0.01.
- Sample size of animal=3, which I doubt is enough for statistical analysis, minimum should have been at least 6 for each group.
- Kindly justify where is the positive group.
- The authors stated the study demonstrated dose dependent effect, however, I can’t see any dose dependent data. Kindly justify.
- Lack of labeling in Figure 4.

 

**Other comments:**

- Tukey’s test is to compare all group, however, the way the author presented was more to Dunnet, which was compared to Control only

Is the work clearly and accurately presented and does it cite the current literature?

Partly

If applicable, is the statistical analysis and its interpretation appropriate?

No

Are all the source data underlying the results available to ensure full reproducibility?

Yes

Is the study design appropriate and is the work technically sound?

Yes

Are the conclusions drawn adequately supported by the results?

Yes

Are sufficient details of methods and analysis provided to allow replication by others?

Partly

Reviewer Expertise:

Natural products, Complementary and alternative medicine, vascular biology, cancer biology, non-communicable diseases.

I confirm that I have read this submission and believe that I have an appropriate level of expertise to confirm that it is of an acceptable scientific standard, however I have significant reservations, as outlined above.

# Response

**Published:** 2020-08-04

**Authors:** NORHAYATI AHMAD

**Affiliations:** UNIVERSITI BRUNEI DARUSSALAM, Brunei

**Section: Introduction**

- Problem statement not clear. 

*Response: Many thanks to the reviewer for kindly reviewing our manuscript. We have revised the introduction by adding the sentences below:*

- *Although V. pinnata is a popular ethnomedicinal plant, scientific studies are lacking to validate its pharmacological properties. It is not clear if this plant has antioxidant, antimicrobial and wound healing properties.*
- *This study is interested to determine if V. pinnata could be a good source of antioxidants.  *
- *Therefore, this study also aims to screen its antibacterial activity against several bacterial strains.*

**Section: Methods**

- Suggest to add another section on “chemicals & reagents”, the authors should list all the chemicals and reagents used in this study by clearly stating the brand, company, and country. 

*Response: We have added a subsection titled “Chemicals and reagents”.*

**Section: Plant materials and preparation of extracts**

- Kindly provide a voucher specimen of the plant.

*Response: We have revised the manuscript by adding: “Voucher specimen of V. pinnata (catalog ID S00059) is available in UBD Herbarium (http://ubdherbarium.fos.ubd.edu.bn/).”*

**Section: Results and discussion**

- Kindly provide yield results.

*Response: We have added a subsection titled “Extraction yields”.*

 

- Caption for all the results (Table and Figure incomplete).

*Response: We have added the caption “Measurement of wound area” for Figure 1.*

**Section: Results and discussion – Antioxidant activities**

- Authors stated and cited phenolic content increased with the increasing polarity of solvent and extraction of total flavonoids significantly increased in polar solvents, which might contribute to the antioxidant activity by Barchan *et al.*, 2014 and Zhang, 2015. However, in the current study ethyl acetate showed to have higher antioxidant activities compared to methanol where methanol polarity is higher than the ethyl acetate. Kindly justify this.

*Response: We have revised the manuscript by adding: “However, in this study, ethyl acetate had higher antioxidant activity compared to the more polar methanol. This could probably mean that certain non-polar compounds, which probably dissolved better in ethyl acetate, might also contribute to the higher antioxidant activity.*

**Section: Results and discussion – Antibacterial activity**

- The authors should include the results for a positive control (reference drug), as it enables a comparison with the other treatment group.

*Response: The results for the positive control is included but one has to click Table 2 and underneath the table, one will see “positive control (streptomycin sulphate) showed inhibition zones ranging from ~20 to 30 mm”.*

- In addition, there is no statistical analysis provided.

*Response: We prefer not to provide statistical analysis as this is only meant as a screening test. Further method such as broth microdilution method has to be carried out to determine which extract is more potent in its antibacterial activity.*

- The concentration of the reference drug should be provided.

*Response: We have added “20 mg/ml” in the methods.*

**

**Section: Results and discussion – Wound healing**

- Kindly provide details of the figures and tables, such as SEM or SD.

*Response*

*The ± SEM values have been included in the respective legends according to the data set.*

 

- Kindly re-run the statistical analysis for the data of Figure 3 (especially Day 14) as the Control group showed big SEM or SD, however, there was a significant difference comparing Control and High dose at P<0.01.

*Response*

*We have rectified the error on our part in the initial figure which was during drawing of the error bars. According to Table 3, the +/- SEM was 0.3. This has been rectified in the latest version of Figure 3.*

 

- Sample size of animal=3, which I doubt is enough for statistical analysis, minimum should have been at least 6 for each group.

*Response*

*We have not conducted power analysis to determine the minimal number of animals for the study and have used the number of animals based on previous reported studies of similar nature. We agree that having more animals per group would produce more sound statistical outcomes. We agree that this is a major shortcoming on our study. However we do feel that the results of our findings are noteworthy.*

 

- Kindly justify where is the positive group.

*Response*

*In this study we have not included a positive control group. The high dose treated animals showed a higher percentage of wound contraction at Day 14 compared to the normal untreated and group and have shown a significant difference.*

 

- The authors stated the study demonstrated dose dependent effect; however, I can’t see any dose dependent data. Kindly justify. 

*Response*

*The dose dependent effect in the context of our work is the effects that we have observed for the high dose (50% (w/w) leaf extracts and low dose group (10% (w/w) leaf extracts where there is a difference in the percentage contractions of the wound.*

 

- Lack of labeling in Figure 4.

*Response*

*A labelled figure is now provided.*

**Other comments:**

- Tukey’s test is to compare all group, however, the way the author presented was more to Dunnet, which was compared to Control only

*Response*

*The study analysis was conducted to compare the wound contractions of treated vs control.*
